# Enhanced Oral Bioavailability of Resveratrol by Using Neutralized Eudragit E Solid Dispersion Prepared via Spray Drying

**DOI:** 10.3390/antiox10010090

**Published:** 2021-01-11

**Authors:** Eun-Sol Ha, Du Hyung Choi, In-hwan Baek, Heejun Park, Min-Soo Kim

**Affiliations:** 1 College of Pharmacy, Pusan National University, 63 Busandaehak-ro, Geumjeong-gu, Busan 46241, Korea; edel@pusan.ac.kr; 2 Department of Pharmaceutical Engineering, Inje University, Gyeongnam 50834, Korea; choidh@inje.ac.kr; 3 College of Pharmacy, Kyungsung University, 309, Suyeong-ro, Nam-gu, Busan 48434, Korea; baek@ks.ac.kr; 4 College of Pharmacy, Duksung Women’s University, 33, Samyangro 144-gil, Dobong-gu, Seoul 01369, Korea; heejunpark@duksung.ac.kr

**Keywords:** bioavailability, solid dispersion, resveratrol, solubility

## Abstract

In this study, we designed amorphous solid dispersions based on Eudragit E/HCl (neutralized Eudragit E using hydrochloric acid) to maximize the dissolution of *trans*-resveratrol. Solid-state characterization of amorphous solid dispersions of *trans*-resveratrol was performed using powder X-ray diffraction, scanning electron microscopy, and particle size measurements. In addition, an in vitro dissolution study and an in vivo pharmacokinetic study in rats were carried out. Among the tested polymers, Eudragit E/HCl was the most effective solid dispersion for the solubilization of *trans*-resveratrol. Eudragit E/HCl significantly inhibited the precipitation of *trans*-resveratrol in a pH 1.2 dissolution medium in a dose-dependent manner. The amorphous Eudragit E/HCl solid dispersion at a *trans*-resveratrol/polymer ratio of 10/90 exhibited a high degree of supersaturation without *trans*-resveratrol precipitation for at least 48 h by the formation of Eudragit E/HCl micelles. In rats, the absolute oral bioavailability (F%) of *trans*-resveratrol from Eudragit E/HCl solid dispersion (10/90) was estimated to be 40%. Therefore, *trans*-resveratrol-loaded Eudragit E/HCl solid dispersions prepared by spray drying offer a promising formulation strategy with high oral bioavailability for developing high-quality health supplements, nutraceutical, and pharmaceutical products.

## 1. Introduction

*Trans*-resveratrol is an antioxidant phytochemical obtained from grapes, berries, and peanuts. The intake of *trans*-resveratrol from dietary supplements and red wine has been shown to have several therapeutic properties [1,2]. Most of the clinical trials involving resveratrol have focused on cancer (prostate, breast, and colorectal), neurological disorders (Alzheimer’s disease and ischemic stroke), cardiovascular diseases (coronary artery disease, atherosclerosis, hypertension, and oxidative stress), diabetes (type 2 and impaired glucose tolerance), and non-alcoholic fatty liver disease [3]. Unfortunately, the use of *trans*-resveratrol by oral administration has been limited by its poor water solubility (below 60 μg/mL in the pH range of 1.2–6.8), instability (conversion of *trans*-resveratrol to *cis*-resveratrol, chemical degradation by alkalizers, and ultraviolet light), and rapid metabolism in the liver, impeding the efficacy of its therapeutic benefits [4,5,6,7]. In an attempt to improve the oral bioavailability of *trans*-resveratrol, several different strategies such as cyclodextrin complexes, cocrystals, liposomes, composite nanoparticles, solid dispersions, solid lipid nanoparticles, microemulsions, self-microemulsifying drug delivery systems, polymeric micelles, nanosuspensions, and nanocrystals have been used [8,9,10,11,12,13,14,15,16,17,18]. Most of these previous attempts have added a large amount of surfactant to the formulation. The absorption is typically higher for the formulations that use a lot of surfactants compared with that of the raw drug material [17,18], but the higher risk of side effects could be resulted in when taken for a long period of time. Thus, it is necessary to develop a formulation that uses less surfactant. We recently developed composite nanoparticles and pure *trans*-resveratrol nanoparticles with high bioavailability using a supercritical antisolvent (SAS) process [19,20]. Furthermore, we demonstrated that the oral bioavailability of *trans*-resveratrol can be increased by improving the dissolution rate and supersaturation of *trans*-resveratrol in an in vitro dissolution test. Regrettably, the mass production of nanoparticles using the SAS process is difficult due to the high cost of high-pressure equipment at a commercial scale of production [21].

In the present study, we hypothesize that an increase in supersaturation of a *trans*-resveratrol solution based on amorphous solid dispersions translates to higher oral bioavailability. Therefore, we designed amorphous solid dispersions using Eudragit E/HCl (Eudragit E neutralized using hydrochloric acid) to maximize the dissolution of *trans*-resveratrol. Eudragit E, a cationic copolymer based on methacrylate, dissolves under conditions with pH < 5.0, while neutralized Eudragit E can be dissolved under intestinal conditions (pH 6.8–8.0) as well as acidic conditions [22,23,24]. Spray drying, commonly used in the preparation of numerous commercialized products, was used to produce amorphous solid dispersions [25,26,27,28]. Besides Eudragit E/HCl, other amorphous solid dispersions of *trans*-resveratrol were produced using different hydrophilic polymers for comparison purposes. Solid-state characterization of amorphous solid dispersions of *trans*-resveratrol was performed using powder X-ray diffraction (PXRD), scanning electron microscopy (SEM), and particle size measurements. Furthermore, an in vitro dissolution test in non-sink conditions and an in vivo oral bioavailability study in rats were carried out to compare different solid dispersions manufactured using the spray drying process.

## 2. Materials and Methods

### 2.1. Materials

*Trans*-resveratrol was provided by Ningbo Liwah Pharmaceutical Co., Ltd. (Ningbo, China). Eudragit E PO (powder form of a cationic copolymer based on dimethylaminoethyl methacrylate, butyl methacrylate, and methyl methacrylate with a ratio of 2:1:1), polyvinylpyrrolidone K30 (PVP K30), and polyvinylpyrrolidone vinyl acetate 64 (PVP VA 64) were kindly donated by BASF (Ludwigshafen, Germany). Hydroxylpropylmethyl cellulose (HPMC 6 cp) and hydroxylpropyl cellulose (HPC-L) were kindly donated by Shin-Etsu Chemical Co., Ltd. (Tokyo, Japan), and Nippon Soda Co., Ltd. (Tokyo, Japan), respectively. Eudragit E/HCl (neutralized Eudragit E with hydrochloric acid) powder was prepared as previously reported [29]. All organic solvents of high-performance liquid chromatography (HPLC) grade were purchased from J.T. Baker (Phillipsburg, NJ, USA). All reagents used were of analytical grade and purchased from Sigma-Aldrich Co., Ltd. (St Louis, MO, USA) or Daejung Chemicals and Metals Co., Ltd. (Siheung-si, Korea).

### 2.2. Preparation of Trans-Resveratrol Solid Dispersion Using the Spray Drying Process

*Trans*-resveratrol solid dispersions with different polymers at various ratios were produced using a B-191 laboratory-scale spray dryer (Buchi, Flawil, Switzerland). The polymers (Eudragit E/HCl, HPC-L, PVP K30, and PVP VA64) and *trans*-resveratrol were dissolved in ethanol at a concentration of 3% *w/v* while HPMC was dissolved in a 70% ethanol solution. The solutions were delivered into the inner line of a two-fluid nozzle at a constant flow rate of 4–6 mL/min using a peristaltic pump. The nozzle’s outer line was supplied with atomization air pressure regulated to 5 kPa, and the solution and drying air were in the same flow direction. Spray drying was carried out in a drying room with an inlet temperature of 80–100 °C and an outlet temperature of 55–70 °C. The prepared powder was obtained in a particle collection chamber equipped with a cyclone.

### 2.3. Morphology Observation and Particle Size Measurement

The morphology of solid dispersion particles was observed using a HITACHI S3500N scanning electron microscope (SEM, Tokyo, Japan). The sample was placed on a brass stub with double-sided electrically conductive adhesive tape and coated with platinum for 60 s under vacuum using an ion sputter to provide electrical conductivity. The image was acquired at a beam acceleration voltage of 30 kV and 1500 times magnification. The particle size and distribution of the spray-dried samples were determined using a HELOS laser diffraction analyzer (Sympatec GmbH, Clausthal-Zellerfeld, Germany). The powder dispersion was made uniform using a RODOS vibrating trough disperser at an air pressure of 0.1 MPa and 5.3 kPa vacuum conditions.

### 2.4. HPLC Analysis

The *trans*-resveratrol content incorporated with the solid dispersion particles was calculated by HPLC analysis of sample solutions prepared by dissolving the solid dispersion particles in ethanol or a 70% ethanol solution. HPLC analysis was conducted using an Agilent 1260 Infinity HPLC system (Agilent Technologies, Santa Clara, CA, USA). A Gemini C18 reversed-phase column (Phenomenex, 150 × 4.6 mm, 5 μm) was used for chromatographic separation using a 40% acetonitrile solution as the mobile phase at a constant flow rate of 0.8 mL/min at 30 °C. Samples of 10 μL were injected and detected at a wavelength of 303 nm.

### 2.5. Powder X-ray Diffraction (PXRD)

The conversion to amorphous solid dispersions was assessed by recording the PXRD patterns of the solid dispersion particles using an Xpert 3 X-ray diffractometer (PHILIPS, Almelo, The Netherlands) at a 1°/min scan rate in the 2θ range of 5–50°.

### 2.6. Dissolution Test

To assess the enhanced dissolution property of *trans*-resveratrol, solid dispersion particles equivalent to 200 mg of *trans*-resveratrol were placed into 500 mL of a pH 1.2 or pH 6.8 dissolution medium at 37 ± 0.5 °C, with a paddle rotation of 100 rpm using a dissolution system (Model 708-DS, Agilent Technologies, Santa Clara, CA, USA). At 0.083, 0.167, 0.25, 0.5, 1, 2, 3, 4, 6, and 24 h post-dissolution test, 3 mL samples were drawn from the vessels and passed through 0.22 μm syringe filters. HPLC analysis was conducted following dilution of the filtered samples with methanol. All sample tests were repeated four times (Tables S1–S4, Supplementary Materials).

### 2.7. Dynamic Light Scattering Measurement

Dynamic light scattering (DLS) measurements were conducted using an ELSZ-1000 particle analyzer (Otsuka Electronics, Tokyo, Japan), according to the manufacturer’s instructions (a scattering angle of 165°), to evaluate the particle size of the sample solutions obtained from the dissolution test. Each sample was measured in triplicate using a He-Ne laser light source (666 nm).

### 2.8. Bioavailability Study in Rats

To evaluate the oral bioavailability of *trans*-resveratrol solid dispersion particles, the in vivo pharmacokinetics of *trans*-resveratrol were investigated using male Sprague-Dawley (SD) rats (200 ± 10 g; Hyochang Science, Daegu, Korea). The animal study protocol complied with the institutional guidelines for the care and use of laboratory animals and was approved by the ethics committee of Kyungsung University (No. 20-017A). Four groups of the rats (five rats per group) were each orally administered via gastric gavage any one of the *trans*-resveratrol raw material, HPMC solid dispersion (10/90), Eudragit E solid dispersion (25/75), and Eudragit E/HCl solid dispersion (10/90) at a dose of 20 mg/kg as *trans*-resveratrol via dispersion state in 1 mL of water before dosing. Blood samples were collected from the jugular vein into heparinized tubes at predetermined time points of prior administration (0 h), 0.25, 0.5, 0.75, 1, 1.5, 2, 4, 6, and 8 h after oral administration. The plasma was separated from the blood samples immediately by centrifugation for 10 min at 6010× *g*. HPLC analysis was used to determine the plasma concentrations of *trans*-resveratrol as previously described [30]. The area under the plasma concentration-time curve (AUC_0→8h_) was estimated based on the linear trapezoidal rule. The maximum *trans*-resveratrol concentration in plasma (C_max_) and the time to reach C_max_ (T_max_) were determined directly from the individual datum.

### 2.9. Statistical Analysis

The differences among the groups were determined using one-way analysis of variance (ANOVA), and post hoc mean separation was conducted using the least-squares difference (LSD) or Student-Newman-Keuls (SNK) tests. Analyses were performed using SPSS 25.0 software (IBM SPSS Statistics, IBM Corporation, Armonk, NY, USA).

## 3. Results and Discussion

**Figure 1:**
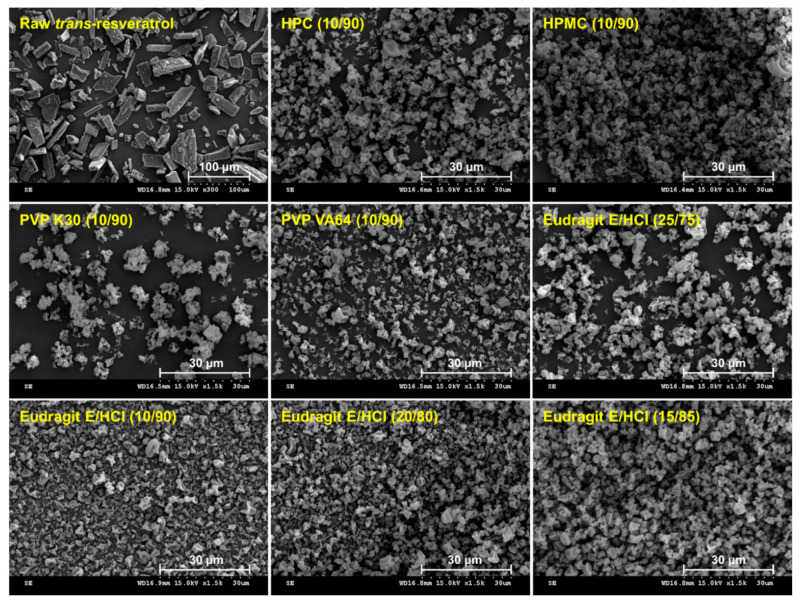
Scanning electron microscope images of raw *trans*-resveratrol and solid dispersion powders produced using the spray drying process.

**Figure 2:**
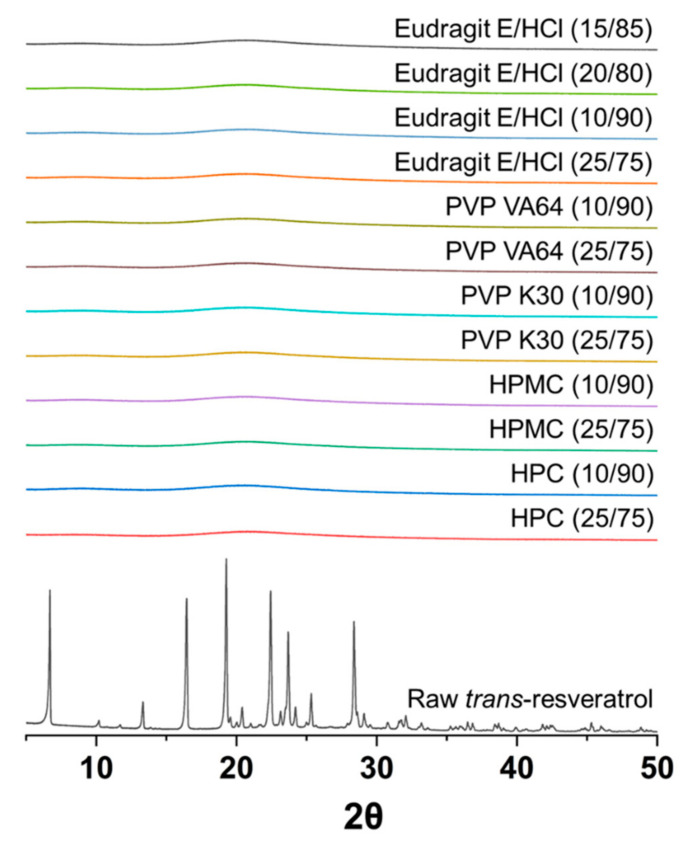
Powder X-ray diffraction patterns of raw *trans*-resveratrol and solid dispersion powders produced using the spray drying process.

*Trans*-resveratrol solid dispersion powders with Eudragit E/HCl, HPMC, HPC, PVP, or PVP VA64 were prepared via spray drying. *Trans*-resveratrol was successfully dispersed into the solid dispersion powders as the encapsulation efficiency was above 96% in all formulations (Table 1). Furthermore, the process yield was above 75% for all formulations. As shown in Figure 1, the raw morphology is needle-shaped, with a mean particle size of 52.5 μm, and the solid dispersion powders were observed as irregularly shaped microparticles. Samples of raw *trans*-resveratrol and solid dispersion powders were analyzed using PXRD to assess the production of amorphous solid dispersions of *trans*-resveratrol (Figure 2). Raw *trans*-resveratrol showed characteristic peaks at 2θ, similar to previously reported data [16]. However, the characteristic crystalline peaks of *trans*-resveratrol were not detected for all solid dispersion powders prepared, indicating amorphous *trans*-resveratrol in the solid dispersions.

### 3.1. Dissolution Data

**Figure 3:**
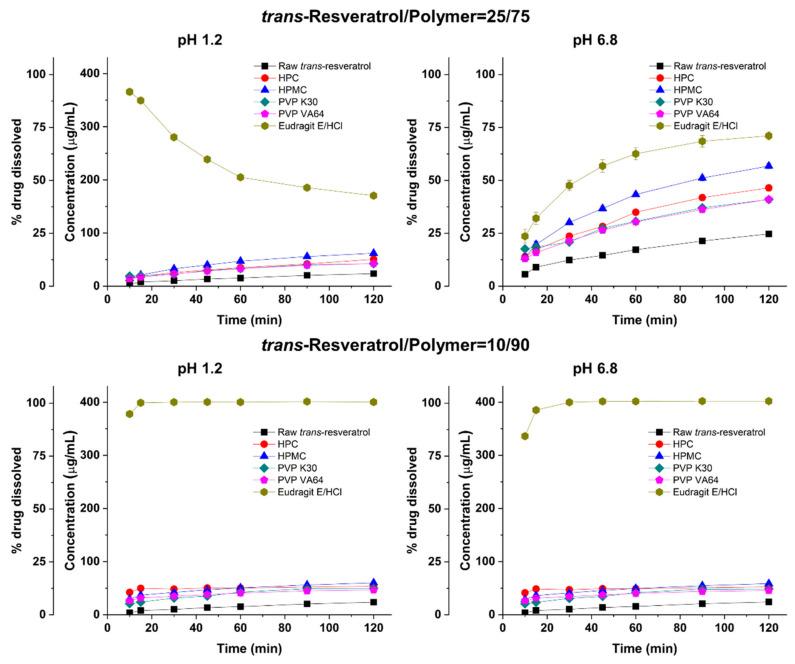
Dissolution profiles of raw *trans*-resveratrol and solid dispersion powders produced using the spray drying process in pH 1.2 and 6.8 dissolution media.

The enhanced dissolution properties of *trans*-resveratrol by amorphous solid dispersions were investigated using dissolution studies in pH 1.2 or pH 6.8 dissolution medium (non-sink condition). As shown in Figure 3, raw *trans*-resveratrol exhibited a slow rate and low degree of dissolution in pH 1.2 and 6.8 dissolution media because of its low solubility at pH 1.2 (52.3 μg/mL) and pH 6.8 (51.1 μg/mL) at 37 °C [19]. At a *trans*-resveratrol/polymer ratio of 25/75, Eudragit E/HCl showed a rapid dissolution rate, a maximum concentration of 365 μg/mL, and gradual subsequent precipitation of *trans*-resveratrol in the pH 1.2 dissolution medium. The concentration of *trans*-resveratrol at 120 min ranked by the SNK test increased in the following order: Eudragit E/HCl solid dispersion > HPMC solid dispersion > HPC solid dispersion > PVP solid dispersion, and PVP VA solid dispersion > raw *trans*-resveratrol. The dissolution data at pH 6.8 showed a similar trend to that at pH 1.2. Generally, the supersaturation and dissolution of poorly water-soluble compounds were enhanced by increasing the hydrophilic polymer amount in the amorphous solid dispersions [31,32,33,34,35]. As shown in Figure 3, dissolution of *trans*-resveratrol was not significantly improved at a *trans*-resveratrol/polymer ratio of 10/90 compared with the other polymers, except Eudragit E/HCl. However, *trans*-resveratrol from the Eudragit E/HCl solid dispersion at a 10/90 ratio was completely dissolved within 30 min in pH 1.2 and pH 6.8 dissolution media. In addition, the precipitation of *trans*-resveratrol did not occur until at least 2 h in pH 1.2 and 6 h in pH 6.8 dissolution media. Among the tested polymers, Eudragit E/HCl was the most effective solid dispersion for the solubilization of *trans*-resveratrol.

Generally, the supersaturated state acquired through amorphous solid dispersion in the dissolution medium is thermodynamically unstable, and subsequent return to the thermodynamically stable state occurs via precipitation [36,37,38,39]. The degree of supersaturation and inhibition of the precipitation of poorly water-soluble compounds can be controlled by using a hydrophilic polymer [40,41]. The polymer acts as a precipitation inhibitor that inhibits crystal growth by blocking the active surface or forming specific interactions with poorly water-soluble compounds. Previously, Wegiel and co-workers reported on the specific interactions between *trans*-resveratrol and polymers by performing spectroscopic analysis [42]. They observed that the increasing order of strength of the hydrogen bonds (the OH stretching region: 3350–2750 cm^−1^) was in the order: PVP > HPMC > Eudragit E, while the ionic interactions related to *trans*-resveratrol crystallization inhibiting performance increased as follows: Eudragit E > PVP > HPMC. Thus, it was suggested that the high degree of supersaturation with an extended supersaturation observed with Eudragit E/HCl might be due to the hydrogen bonding and ionic interactions between *trans*-resveratrol and Eudragit E.

**Figure 4:**
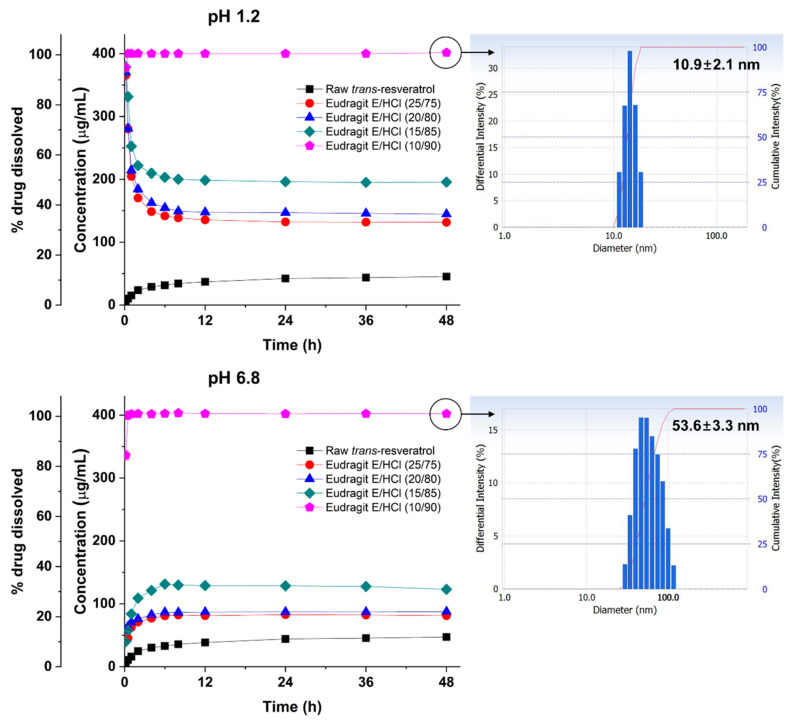
Effect of Eudragit E/HCl ratio on the dissolution of *trans*-resveratrol from solid dispersions in pH 1.2 and 6.8 dissolution media.

To further investigate the effect of Eudragit E/HCl on the dissolution of *trans*-resveratrol, amorphous solid dispersions were prepared at different *trans*-resveratrol/Eudragit E/HCl ratios of 25/75, 20/80, 15/85, and 10/90 and evaluated for 48 h in a pH 1.2 or pH 6.8 dissolution medium (non-sink condition). As shown in Figure 4, a high degree of supersaturation was observed within 30 min at all ratios, and Eudragit E/HCl significantly inhibited the precipitation of *trans*-resveratrol in the pH 1.2 dissolution medium in a dose-dependent manner. However, the degree of supersaturation of *trans*-resveratrol was enhanced by Eudragit E/HCl in the pH 6.8 dissolution medium in a dose-dependent manner. Interestingly, *trans*-resveratrol from the Eudragit E/HCl solid dispersion at a 10/90 ratio was completely dissolved within 30 min, and precipitation did not occur until at least 48 h in both the pH 1.2 and pH 6.8 dissolution media. At this time, DLS measurements were carried out to evaluate the soluble state of *trans*-resveratrol. As depicted in Figure 4, the solution’s mean particle size was 10.9 nm for pH 1.2 and 53.6 nm for pH 6.8. Yoshida and co-workers demonstrated micelle formation at Eudragit E/HCl concentrations above 30 μg/mL, and the particle size of tacrolimus-loaded Eudragit E/HCl micelles varied from 10 to 369.8 nm in different medium conditions [22,24]. Recently, Lin and co-workers reported the solubilization of ibuprofen, felodipine, and bifendate by Eudragit E in HCl solution via micelle formation [43]. In fact, the high solubilization of *trans*-resveratrol by Eudragit E/HCl solid dispersion prepared by the spray drying process might be used to estimate the formation of Eudragit E/HCl micelles, similar to previously reported poorly water-soluble compounds.

### 3.2. Oral Bioavailability in SD Rats

**Figure 5:**
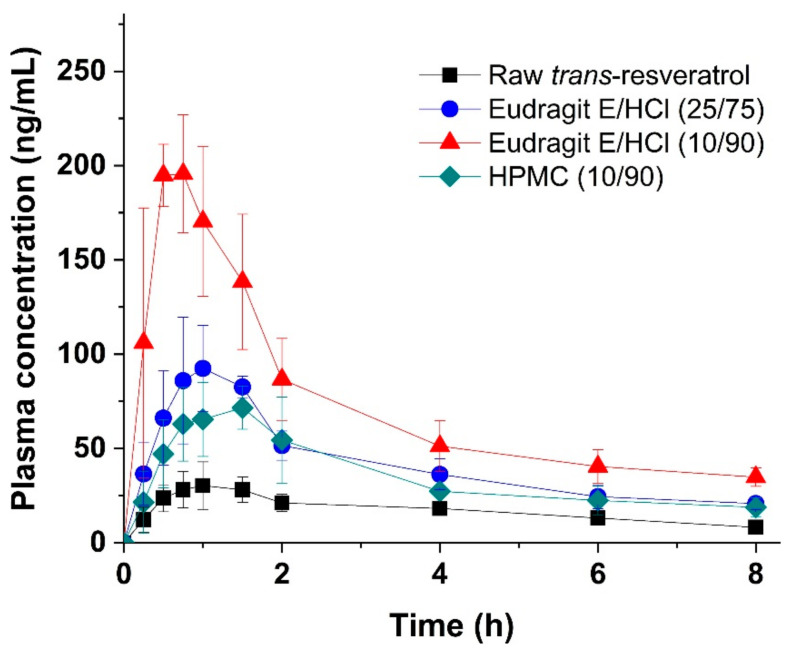
Plasma concentration-time profiles of raw *trans*-resveratrol and solid dispersion powders produced using the spray drying process after oral administration in Sprague-Dawley rats.

The effects of the observed degree and duration of in vitro supersaturation on *trans*-resveratrol bioavailability were further investigated by the determination of in vivo pharmacokinetic parameters using oral administration of raw *trans*-resveratrol, HPMC solid dispersion (10/90), and Eudragit E/HCl solid dispersion at 25/75 and 10/90 ratios to SD rats (Table 2). Furthermore, the absolute oral bioavailability (F%) of *trans*-resveratrol was estimated by dividing the AUC_0→8 h_ value obtained from oral administration by the AUC_0→8 h_ value obtained from intravenous administration with dose normalization. The AUC_0→8 h_ value of *trans*-resveratrol after intravenous administration used was from our previous study [20]. As shown in Figure 5, the oral absorption of *trans*-resveratrol was significantly enhanced by the Eudragit E/HCl solid dispersion. In addition, the Eudragit E/HCl solid dispersion (10/90) was 4.2- and 5.5-fold higher in AUC_0→8 h_ and C_max_ values respectively, than raw *trans*-resveratrol. Based on the ANOVA, there were significant differences among the solid dispersions (*p* < 0.05), which, in order of increasing pharmacokinetic parameters, were ranked by the SNK test as follows: raw *trans*-resveratrol < HPMC solid dispersion (10/90) = Eudragit E/HCl solid dispersion (25/75) < Eudragit E/HCl solid dispersion (10/90) for AUC_0→8 h_ values, and raw *trans*-resveratrol < HPMC solid dispersion (10/90) < Eudragit E/HCl solid dispersion (25/75) < Eudragit E/HCl solid dispersion (10/90) for C_max_ values. In fact, the AUC_0→8 h_ and C_max_ values of *trans*-resveratrol increased with the increasing degree and duration of in vitro supersaturation. This tendency is similar to that of *trans*-resveratrol-loaded composite nanoparticles previously reported [20,21]. Furthermore, the absolute oral bioavailability (F%) of *trans*-resveratrol was estimated to be 40% for the Eudragit E/HCl solid dispersion (10/90). Previously, it was revealed that the absolute oral bioavailability (F%) of *trans*-resveratrol in rats varied between 2.6% and 46.4% in different solubilizing formulation systems [44,45,46,47]. Thus, Eudragit E/HCl solid dispersion prepared by spray drying is an effective oral formulation with high absolute bioavailability for poorly water-soluble *trans*-resveratrol.

## 4. Conclusions

In the present study, we presented an amorphous solid dispersion of *trans*-resveratrol through a Eudragit E/HCl solid dispersion prepared by a spray drying process. The amorphous Eudragit E/HCl solid dispersion at a *trans*-resveratrol/polymer ratio of 10/90 exhibited a high degree of supersaturation without precipitation of *trans*-resveratrol for at least 48 h by the formation of Eudragit E/HCl micelles. In SD rats, the absolute oral bioavailability (F%) of *trans*-resveratrol from the Eudragit E/HCl solid dispersion (10/90) was estimated to be 40%. Therefore, *trans*-resveratrol-loaded Eudragit E/HCl solid dispersions prepared by spray drying offer a promising formulation strategy with high oral bioavailability for developing high-quality health supplements, nutraceutical, and pharmaceutical products.

## Figures and Tables

**Table 1:** Encapsulation efficiency and particle size of raw *trans*-resveratrol and solid dispersion powders produced using the spray drying process.

Solid Dispersions (*Trans*-Resveratrol/Polymer Ratio)	Encapsulation Efficiency ^a^ (%)	Volume Mean Particle Size ^a^ (μm)
HPC (25/75)	97.7 ± 1.5	4.84 ± 0.31 (1.89) ^b^
HPC (10/90)	96.9 ± 2.1	4.12 ± 0.23 (1.83)
HPMC (25/75)	98.9 ± 1.4	5.23 ± 0.31 (1.93)
HPMC (10/90)	98.3 ± 1.1	4.98 ± 0.22 (1.81)
PVP K30 (25/75)	99.1 ± 1.0	4.03 ± 0.21 (1.71)
PVP K30 (10/90)	98.2 ± 0.9	3.98 ± 0.19 (1.65)
PVP VA64 (25/75)	98.6 ± 1.2	3.69 ± 0.32 (1.65)
PVP VA64 (10/90)	98.8 ± 1.1	3.98 ± 0.19 (1.69)
Eudragit E/HCl (25/75)	98.2 ± 0.8	3.76 ± 0.29 (1.53)
Eudragit E/HCl (10/90)	98.0 ± 0.5	3.92 ± 0.27 (1.62)
Eudragit E/HCl (20/80)	98.3 ± 0.9	3.89 ± 0.15 (1.71)
Eudragit E/HCl (15/85)	98.6 ± 0.6	4.01 ± 0.32 (1.65)
Raw trans-resveratrol	-	52.5 ± 1.32 (2.21)

^a^ Mean ± standard deviation (*n* = 4). ^b^ The span is the width of the distribution and is calculated as follows (*d*_90_ − *d*_10_)/*d*_50_, where *d*_10_, *d*_50_, and *d*_90_ are the diameter sizes at 10%, 50%, and 90% in a cumulative size distribution, respectively.

**Table 2:** Pharmacokinetic parameters of *trans*-resveratrol in rats after oral administration of raw *trans*-resveratrol or solid dispersions.

Solid Dispersions (*Trans*-Resveratrol/Polymer Ratio)	AUC_0→8h_ (ng·h/mL)	C_max_ (ng/mL)	T_max_ (h)	F (%)
Eudragit E/HCl (25/75)	330.0 ± 41.8 ^a^	109.6 ± 22.4 ^a,b^	1.0 ± 0.3	22.6
Eudragit E/HCl (10/90)	583.9 ± 92.1 ^a,b,c^	204.4 ± 25.5 ^a,b,c^	0.8 ± 0.2	40.0
HPMC (10/90)	279.8 ± 40.7 ^a^	79.6 ± 11.2 ^a^	1.1 ± 0.4	19.2
Raw *trans*-resveratrol	138.9 ± 22.0	37.0 ± 7.7	1.2 ± 0.3	9.5

Note: ^a^ *p* < 0.05 vs. raw *trans*-resveratrol; ^b^ *p* < 0.05 vs. HPMC solid dispersion (10/90) ^c^ *p* < 0.05 vs. Eudragit E/HCl solid dispersion (25/75). Mean ± standard deviation (*n* = 5). AUC_0→8 h_, the area under the plasma concentration versus time curve; C_max_, the maximum plasma concentration of *trans*-resveratrol; T_max_, the time required to reach Cmax; F (%), the absolute oral bioavailability.

## Data Availability

The data presented in this study are available on request from the corresponding author.

## Supplementary Materials

The following are available online at https://www.mdpi.com/2076-3921/10/1/90/s1, Table S1: Dissolution data (concentration, μg/mL) of raw trans-resveratrol and solid dispersion powders produced using the spray drying process in pH 1.2 dissolution media, Table S2: Dissolution data (concentration, μg/mL) of raw trans-resveratrol and solid dispersion powders produced using the spray drying process in pH 6.8 dissolution media, Table S3: Dissolution data (concentration, μg/mL) of raw trans-resveratrol and Eudragit E/HCl solid dispersion powders produced using the spray drying process in pH 1.2 dissolution media, Table S4: Dissolution data (concentration, μg/mL) of raw trans-resveratrol and Eudragit E/HCl solid dispersion powders produced using the spray drying process in pH 6.8 dissolution media.
