# Supplementary material for: Enhanced Oral Bioavailability of Resveratrol by Using Neutralized Eudragit E Solid Dispersion Prepared via Spray Drying
Source: Antioxidants (Basel). 2021 Jan 11;10(1):90. doi: 10.3390/antiox10010090 (PMC7828062; doi:10.3390/antiox10010090)
Supplement: Supplementary file 1 [file antioxidants-10-00090-s001.pdf]

antioxidants

MDPI

Enhanced Oral Bioavailability of Resveratrol by Using Neutralized Eudragit E Solid Dispersion Prepared via Spray Drying

Eun-Sol Ha [1], Du Hyung Choi [2], In-hwan Baek [3], Heejun Park [4] and Min-Soo Kim [1,*]

Table S1. Dissolution data (concentration, μg/mL) of raw *trans*-resveratrol and solid dispersion powders produced using the spray drying process in pH 1.2 dissolution media.

Solid dispersions (*trans*-resveratrol/polymer ratio)	0.167 h	0.25 h	0.5 h	0.75 h	1 h	1.5 h	2 h
HPC (25/75)	14.9 ± 1.2[a]	18.5 ± 1.7	25.5 ± 0.5	30.6 ± 0.8	34.9 ± 1.3	41.9 ± 1.4	50.6 ± 1.5
HPC (10/90)	42.2 ± 1.6	49.7 ± 1.7	48.3 ± 0.5	50.4 ± 0.9	50.3 ± 1.3	52.2 ± 1.4	53.8 ± 1.5
HPMC (25/75)	14.6 ± 1.8	21.2 ± 1.4	32.7 ± 0.2	39.9 ± 0.9	47.1 ± 1.1	55.7 ± 1.3	61.9 ± 1.3
HPMC (10/90)	29.5 ± 1.7	36.9 ± 1.5	42.1 ± 0.5	46.9 ± 1.3	50.6 ± 1.2	56.1 ± 1.4	60.1 ± 1.3
PVP K30 (25/75)	18.9 ± 1.4	19.9 ± 1.4	22.5 ± 0.6	29.6 ± 1.5	33.2 ± 1.1	40.2 ± 1.3	42.4 ± 1.0
PVP K30 (10/90)	21.1 ± 1.2	23.8 ± 1.5	31.4 ± 0.8	35.2 ± 1.8	42.6 ± 1.1	49.2 ± 1.3	49.6 ± 0.9
PVP VA64 (25/75)	13.8 ± 0.8	17.0 ± 1.3	23.1 ± 0.9	28.6 ± 1.9	32.9 ± 1.0	39.4 ± 1.2	42.4 ± 0.7
PVP VA64 (10/90)	26.5 ± 1.1	31.6 ± 1.6	34.8 ± 0.8	37.9 ± 1.4	41.0 ± 1.2	45.0 ± 1.4	46.7 ± 1.2
Eudragit E/HCl (25/75)	365.7 ± 6.0	349.3 ± 5.1	280.2 ± 4.4	238.6 ± 5.4	204.9 ± 5.2	185.4 ± 5.0	170.4 ± 3.5
Eudragit E/HCl (10/90)	377.8 ± 4.7	398.8 ± 2.7	400.1 ± 3.0	400.3 ± 2.2	400.1 ± 2.3	401.1 ± 2.7	400.2 ± 2
Raw *trans*-resveratrol	3.8 ± 0.2	7.9 ± 0.3	10.2 ± 0.5	13.4 ± 0.8	15.4 ± 1.1	20.4 ± 1.1	23.6 ± 1.3

[a]Mean ± standard deviation (n=4).

Table S2. Dissolution data (concentration, µg/mL) of raw *trans*-resveratrol and solid dispersion powders produced using the spray drying process in pH 6.8 dissolution media.

**Solid dispersions (*trans*-resveratrol/polymer ratio)**	**0.167 h**	**0.25 h**	**0.5 h**	**0.75 h**	**1 h**	**1.5 h**	**2 h**
HPC (25/75)	13.9 ± 1.6[a]	17.3 ± 1.7	23.6 ± 0.4	28.3 ± 0.9	34.9 ± 1.3	41.9 ± 1.4	46.5 ± 1.5
HPC (10/90)	41.3 ± 1.7	48.6 ± 1.7	47.2 ± 0.6	49.2 ± 1.2	49.1 ± 1.3	51.0 ± 1.5	52.5 ± 1.5
HPMC (25/75)	13.7 ± 1.7	19.7 ± 1.5	30.1 ± 0.4	36.7 ± 1.2	43.3 ± 1.1	51.1 ± 1.4	56.8 ± 1.3
HPMC (10/90)	29.1 ± 1.5	36.2 ± 1.6	41.2 ± 0.8	45.9 ± 1.6	49.5 ± 1.2	54.8 ± 1.5	58.7 ± 1.2
PVP K30 (25/75)	17.6 ± 1.1	18.5 ± 1.5	20.8 ± 0.8	27.3 ± 1.8	30.7 ± 1.1	37.0 ± 1.3	41.0 ± 0.9
PVP K30 (10/90)	20.9 ± 1.2	23.5 ± 1.6	30.8 ± 0.9	34.6 ± 1.7	41.7 ± 1.3	48.0 ± 1.5	48.5 ± 1.2
PVP VA64 (25/75)	13.0 ± 1.0	15.9 ± 1.6	21.4 ± 0.7	26.4 ± 1.4	30.4 ± 1.2	36.2 ± 1.4	41.0 ± 1.1
PVP VA64 (10/90)	26.1 ± 1.5	31.0 ± 1.7	34.2 ± 0.7	37.1 ± 1.4	40.2 ± 1.3	44.0 ± 1.5	45.7 ± 1.3
Eudragit E/HCl (25/75)	23.6 ± 3.3	32.1 ± 2.8	47.6 ± 2.5	56.8 ± 3.0	62.5 ± 2.9	68.5 ± 2.7	71.1 ± 2.0
Eudragit E/HCl (10/90)	336.1 ± 5.6	385.2 ± 4.3	399.8 ± 3.2	401.5 ± 2.1	401.6 ± 2.1	402.0 ± 2.0	402.0 ± 2.2
Raw *trans*-resveratrol	5.6 ± 0.4	9.0 ± 0.5	12.4 ± 0.2	14.6 ± 0.6	17.2 ± 0.9	21.4 ± 1.1	24.7 ± 1.1

[a]Mean ± standard deviation (n=4).

Table S3. Dissolution data (concentration, µg/mL) of raw *trans*-resveratrol and Eudragit E/HCl solid dispersion powders produced using the spray drying process in pH 1.2 dissolution media.

**Solid dispersions (*trans*-resveratrol/polymer ratio)**	**0.167 h**	**0.5 h**	**1 h**	**2 h**	**4 h**	**6 h**	**8 h**	**12 h**	**24 h**	**36 h**	**48 h**
Eudragit E/HCl (25/75)	365.7 ± 7.5[a]	280.2 ± 4.6	204.9 ± 6.5	170.4 ± 3.2	148.7 ± 4.5	141.7 ± 5.3	138.6 ± 3.3	135.6 ± 2.3	132.3 ± 3.4	131.9 ± 3.6	131.6 ± 3.5
Eudragit E/HCl (20/80)	370.3 ± 7.1	281.0 ± 6.5	214.3 ± 5.7	184.4 ± 5.5	162.6 ± 4.2	154.6 ± 3.8	149.3 ± 3.2	147.6 ± 3.3	147.0 ± 3.2	145.8 ± 2.9	144.6 ± 3.0
Eudragit E/HCl (15/85)	378.8 ± 8.6	331.5 ± 6.7	252.6 ± 7.6	221.9 ± 5.7	209.7 ± 7.2	203.2 ± 5.3	200.1 ± 5.3	198.6 ± 5.4	196.2 ± 5.2	195.1 ± 4.9	195.6 ± 4.7
Eudragit E/HCl (10/90)	377.8 ± 6.9	400.1 ± 5.4	400.1 ± 4.2	400.2 ± 3.2	400.0 ± 2.3	400.0 ± 2.2	400.0 ± 2.6	400.0 ± 2.4	400.0 ± 2.5	400.0 ± 2.3	401.6 ± 3.2
Raw *trans*-resveratrol	3.8 ± 0.4	10.2 ± 0.3	15.4 ± 0.5	23.6 ± 0.6	29.0 ± 0.7	31.6 ± 0.8	34.2 ± 0.9	36.9 ± 1.1	42.2 ± 1.2	43.6 ± 1.3	45.2 ± 1.6

[a]Mean ± standard deviation (n=4).

Table S4. Dissolution data (concentration, μg/mL) of raw trans-resveratrol and Eudragit E/HCl solid dispersion powders produced using the spray drying process in pH 6.8 dissolution media.

Solid dispersions (*trans*-resveratrol/polymer ratio)	0.167 h	0.5 h	1 h	2 h	4 h	6 h	8 h	12 h	24 h	36 h	48 h
Eudragit E/HCl (25/75)	17.0 ± 4.1[a]	45.5 ± 2.5	62.5 ± 3.5	71.1 ± 1.8	77.2 ± 2.5	80.9 ± 2.9	82.3 ± 1.9	81.1 ± 1.3	82.8 ± 1.9	82.3 ± 2.0	81.1 ± 2.0
Eudragit E/HCl (20/80)	57.2 ± 3.8	63.8 ± 3.5	70.7 ± 3.1	75.9 ± 3.0	82.6 ± 2.3	86.5 ± 2.1	86.4 ± 1.8	86.9 ± 1.9	87.1 ± 1.8	87.0 ± 1.6	87.5 ± 1.7
Eudragit E/HCl (15/85)	40.1 ± 4.6	57.8 ± 3.6	83.9 ± 4.1	109.0 ± 3.1	121.4 ± 3.9	131.4 ± 2.9	129.9 ± 2.9	129.0 ± 3.0	128.6 ± 2.9	127.7 ± 2.7	123.0 ± 2.6
Eudragit E/HCl (10/90)	336.1 ± 8.0	399.8 ± 6.9	401.6 ± 5.6	402.0 ± 5.4	401.5 ± 4.3	402.3 ± 3.4	403.1 ± 3.6	402.1 ± 3.8	401.9 ± 3.6	402.1 ± 3.5	402.0 ± 3.9
Raw *trans*-resveratrol	4.0 ± 0.5	10.7 ± 0.3	16.1 ± 0.6	24.7 ± 0.9	30.3 ± 0.8	33.0 ± 1.1	35.8 ± 1.2	38.6 ± 1.3	44.2 ± 1.5	45.5 ± 1.6	47.3 ± 1.5

[a]Mean ± standard deviation (n=4).
